# Gender Inequities in Quality of Care among HIV-Positive Individuals Initiating Antiretroviral Treatment in British Columbia, Canada (2000–2010)

**DOI:** 10.1371/journal.pone.0092334

**Published:** 2014-03-18

**Authors:** Allison Carter, Jeong Eun Min, William Chau, Viviane D. Lima, Mary Kestler, Neora Pick, Deborah Money, Julio S G. Montaner, Robert S. Hogg, Angela Kaida

**Affiliations:** 1 Faculty of Health Sciences, Simon Fraser University, Burnaby, British Columbia, Canada; 2 British Columbia Centre for Excellence in HIV/AIDS, Vancouver, British Columbia, Canada; 3 Oak Tree Clinic, BC Women's Hospital and Health Centre, Vancouver, British Columbia, Canada; 4 Women's Health Research Institute, BC Women's Hospital and Health Centre, Vancouver, British Columbia, Canada; 5 Department of Obstetrics and Gynecology, Faculty of Medicine, University of British Columbia, Vancouver, British Columbia, Canada; 6 Department of Medicine, Faculty of Medicine, University of British Columbia, Vancouver, British Columbia, Canada; University of Pittsburgh, United States of America

## Abstract

**Objectives:**

We measured gender differences in “Quality of Care” (QOC) during the first year after initiation of antiretroviral therapy and investigated factors associated with poorer QOC among women.

**Design:**

QOC was estimated using the Programmatic Compliance Score (PCS), a validated metric associated with all-cause mortality, among all patients (≥19 years) who initiated ART in British Columbia, Canada (2000–2010).

**Methods:**

PCS includes six indicators of non-compliance with treatment initiation guidelines at baseline (not having drug resistance testing before treatment; starting on a non-recommended regimen; starting therapy at CD4<200 cells/mm^3^) and during first-year follow-up (receiving <3 CD4 tests; receiving <3 viral load tests; not achieving viral suppression within six months). Summary scores range from 0–6; higher scores indicate poorer QOC. Multivariable ordinal logistic regression was used to measure if female gender was an independent predictor of poorer QOC and factors associated with poorer QOC among women.

**Results:**

QOC was determined for 3,642 patients (20% women). At baseline: 42% of women (34% men) did not have resistance testing before treatment; 17% of women (9% men) started on a non-recommended regimen (all p<0.001). At follow-up: 17% of women (11% men) received <3 CD4; 17% of women (11% men) received <3 VL; 50% of women (41% men) did not achieve viral suppression (all p<0.001). Overall, QOC was better among men (mean PSC = 1.54 (SD = 1.30)) compared with women (mean = 1.89 (SD = 1.37); p<0.001). In the multivariable model, female gender (AOR = 1.16 [95% CI: 0.99–1.35]; p = 0.062) remained associated with poorer QOC after covariate adjustment. Among women, those with injection drug use history, of Aboriginal ancestry, from Vancouver Island, and who initiated ART in earlier years were more likely to have poorer QOC.

**Conclusions:**

Poorer QOC among women, especially from marginalized communities, demands that barriers undermining women's access to high-quality care be addressed to improve treatment and health for women with HIV.

## Introduction

Compliance with HIV clinical care guidelines during the first year after initiation of antiretroviral therapy (ART) is a key predictor of health and survival [1], [2], [3]. In 2012, a new ‘Quality of Care’ (QOC) metric, called the ‘Programmatic Compliance Score’ (PCS), was developed and validated to measure the impact of sub-optimal compliance with treatment initiation guidelines [1]. In this study, non-receipt of care consistent with six quality indicators during the first year on HAART was shown to be strongly associated with morbidity and mortality [1].

This assessment measured QOC for men and women combined, although it is acknowledged that care and health outcomes are strongly influenced by gender. For instance, women are diagnosed at more advanced disease states and have longer delays in initiating ART [4], [5], [6], [7]. While some authors have reported women are less likely than men to achieve virological suppression [8], other evaluations have showed similar [9], [10] or improved [11] virological suppression in women. Nevertheless, women are more likely to be non-adherent, have treatment interruptions, and experience more adverse drug reactions [4], [5], [6], [7], [12], [13]. However, it remains unclear whether this new measure of QOC differs by gender and if there are gender-specific reasons for differential receipt of recommended care.

As women comprise a growing proportion of prevalent and incident HIV cases in Canada and globally [14], [15], the primary objective of this study was to measure gender differences in QOC and to investigate patient- and system-level factors associated with poorer QOC among women within a cohort of HIV-positive individuals initiating HAART in British Columbia (BC), Canada. Identifying and mitigating potential gendered gaps in QOC is critical to inform programming within the ‘Seek and Treat for Optimal Prevention of HIV/AIDS’ (STOP HIV/AIDS) and similar initiatives aimed at expanding access to HIV testing and treatment within the context of ‘Treatment as Prevention’ [16], [17]. Defining the gender gap in care is also crucial to ensure the design of appropriate, accessible, supportive, and inclusive women-centered HIV services that more fully meet the needs of diverse communities of women living with HIV.

## Methods

### Ethical Approval

The Centre's HIV/AIDS Drug Treatment program has received ethical approval from the University of British Columbia Ethics Review Committee at its St. Paul'sHospital site. The program also conforms with the province's Freedom of Information and Protection of Privacy Act.

### Study Population

This study was conducted using population-based data from the Drug Treatment Program (DTP) at the BC Centre for Excellence in HIV/AIDS (BC-CfE), a province-wide health care program that centrally distributes HIV medications free of charge to all people living with HIV and clinically eligible for treatment. The current study population includes all individuals (≥19 years) who initiated ART between January 1, 2000 and September 31, 2010 and had at least one baseline CD4 and viral load test done within six months prior to treatment initiation. Patients who died (n = 184) or moved out of BC (n = 49) within the one year of follow-up were excluded from this analysis. Ethical approval for this study was provided by the Research Ethics Boards of Providence Health Care/University of British Columbia. Patient records were anonymized and de-identified prior to analysis.

### Primary Outcome

The primary outcome was QOC, estimated using the PCS metric which includes six indicators of non-compliance with treatment initiation guidelines recommended by BC-CfE's HIV/AIDS Therapeutic Guideline Committee [18] and closely aligned with those published by the International AIDS Society-USA (IAS-USA) panel [3]. These include three indicators at baseline ((1) not having drug resistance testing prior to starting treatment; (2) starting on a non-recommended ART regimen (according to contemporary guidelines); and (3) starting therapy with CD4 less than 200 cells/mm^3^) and three indicators during the first-year of follow-up ((4) receiving less than 3 CD4 cell count tests; (5) receiving less than 3 plasma viral load tests; and (6) not achieving HIV viral load suppression within six months of treatment initiation). Non-compliance to each clinical guideline is coded as 1, and compliance is coded as 0. Summary scores, therefore, range from 0 (perfect compliance) to 6 (perfect non-compliance). Higher PCS scores indicate poorer QOC, as they represent greater non-receipt of recommended care. Further, poorer QOC is predictive of worse health outcomes. As shown in the validation study, individuals with a PCS score of 4 or higher have a probability of mortality 22 times higher and a probability of an AIDS-defining illness 7 times higher than individuals with a PCS score of 0 [1].

### Laboratory Data

Assessment of receipt of drug resistance testing prior to time of ART initiation is based on HIV genotypic resistance testing data conducted centrally by the St Paul's Hospital virology laboratory. Samples from across the province are tested and assigned to one of four resistance categories based on a modification of the 2011 IAS-USA list of mutations [19].

All plasma viral load measurements are also centrally done at the St Paul's Hospital virology laboratory. The quantification range of plasma viral load assays has evolved over time. Thus, for analytical purposes, lower and upper measures were truncated to range from <50 to >100,000 copies/mL. Viral suppression was defined by two consecutive plasma viral loads <50 copies/mL.

CD4 testing, measured by flow cytometry followed by fluorescent monoclonal antibody analysis, is conducted by several different laboratories across BC. The DTP database captures data on an estimated 80% of all CD4 tests done across the province [1]. Given that our analysis relies on receipt versus non-receipt of CD4 testing (rather than the actual CD4 count), and since it is customary clinical practice to order both CD4 and plasma viral load tests at the same clinical visit, we adjusted the data by replacing number of CD4 tests with number of plasma viral load tests. We tested the validity of this approach by restricting the sample to include only patients from St. Paul's Hospital, which provides CD4 data on every patient in the DTP. We found >99% consistency in reports of viral load and CD4 testing, supporting the approach described above.

### Antiretroviral Regimen

Recommended antiretroviral therapy regimens have undergone four iterations since 2000, based on the BC guidelines for treating HIV-positive adults, as in the IAS-USA guidelines [3], [20], [21], [22], [23], [24]. Therefore, rules were developed to classify prescribed regimes as being appropriate or not as per the guidelines of that time, as described in the Supplementary text (Figure S1).

### Explanatory Variables of Interest

Explanatory variables of interest were obtained from the DTP. Individuals are automatically enrolled in the DTP when they are first prescribed ART. The prescribing physician must complete a drug request form detailing baseline information, such as the patient's address, CD4 cell counts, plasma viral load levels, past HIV-specific drug history, and history of injection drug use (IDU).[25] Aboriginal ancestry has been collected in the DTP over time through program enrolment forms and cohort surveys. As reporting is not mandatory, data on history of IDU and Aboriginal ancestry are unknown for 27% and 49% of all patients, respectively.

Patient factors included in this analysis were gender (female *vs.* male), age at enrolment, history of IDU (yes *vs*. no *vs*. unknown), and Aboriginal ancestry (yes *vs*. no *vs*. unknown). Further, we considered system factors such as prescriber experience (estimated by median HIV patient caseload), year of ART initiation (2000–2003, 2004–2007, *vs*. 2008–2010), and place of residence at ART initiation (Fraser, Interior + Northern, Vancouver Island, *vs.* Vancouver Coastal) [26]. Place of residence was based on BC's five geographically-distinct regional health authorities and was used to control for the heterogeneity in patients' access to treatment and sociodemographic factors not previously defined. Interior and Northern regions were combined due to low sample size.

### Statistical Analyses

We report and compare baseline characteristics of study participants by gender using Pearson χ^2^ test for categorical variables and Wilcoxon rank-sum test for continuous variables. In the bivariate analysis, we compared non-compliance with each of the 6 PCS items by gender using Pearson's χ^2^ test.

Following this, we ran two sets of multivariable ordinal logistic regression analyses: (1) to measure if gender was an independent predictor of poorer QOC after adjusting for confounding factors and (2) to measure factors associated poorer QOC among women. For both analyses, we used a partial proportional odds model since the proportional odds assumption was not valid for some of the variables in the model.

In the first analysis, potential confounders were selected for inclusion in the final models using a backward selection approach, which considered the magnitude of change in the coefficient of the exposure variable. Starting with a model including all available variables, confounding variables were dropped one at a time, using the relative change in the coefficient for gender as a criterion, until the minimum change from the full model exceeded 5%. In the second analysis, a backward stepwise technique based on two criteria (Akaike Information Criterion (AIC) and Type III p-values) was used in the selection of covariates to build this model, with the least significant variable dropped until the final model had the optimum (minimum) AIC [27], [28]. From this analysis, probabilities of having each level of PCS score were estimated among women and were stratified by patient- and system-level characteristics. Patients with unknown place of residence were excluded from both ordinal logistic models due to low sample size. All statistical tests were two-sided and considered significant at α = 0.05. All analyses were conducted using SAS version 9.3 (SAS, North Carolina, United States).

## Results

A total of 3,642 antiretroviral naïve adults were eligible for this study. Of this total, 745 (20%) were women. At baseline, women had a median CD4 cell count of 210 cells/mm^3^ (Interquartile range [IQR]: 130–310 cells/mm^3^) and a median plasma viral load of 4.8 log_10_ copies/mL (Q1–Q3: 4.1–5.0 log_10_ copies/mL), versus men who had a CD4 of 200 cells/mm^3^ (Q1–Q3: 100–300 cells/mm^3^) (p = 0.008) and viral load of 4.9 log_10_ copies/mL (Q1–Q3: 4.5–5.0 log_10_ copies/mL) (p<0.001). The baseline patient- and system-level characteristics of the study population stratified by gender are shown in **Table 1**. Compared with men, women were more likely to be younger (median 36 years [Q1–Q3: 30–44 years] vs. 42 years [Q1–Q3: 36–49 years], p<0.001), of Aboriginal ancestry (25% vs. 9%, p<0.001), and have a history of IDU (58% vs. 33%, p<0.001). At the system-level, women were more likely to be cared for by providers with lower HIV patient caseloads (96 [Q1–Q3: 18–204] vs. 108 [Q1–Q3: 24–266], p<0.001). Further, gender differences also existed by place of residence at the start of ART, with 44% of women living in the Vancouver area compared to 61% of men (p<0.001). No gender differences were observed by year of treatment initiation.

**Table 1:** Baseline characteristics of study population by gender.

	Gender		
	Women (n = 745)	Men (n = 2897)	p-value
**Baseline clinical indicators**			
**Baseline CD4 cell count** (cells/mm^3^)			
Median	210	200	p<0.001
Interquartile Range	130–310	100–300	
**Baseline plasma viral load** (log_10_ copies/mL)			
Median	4.8	4.9	p<0.001
Interquartile Range	4.1–5.0	4.5–5.0	
**Patient characteristics**			
**Age**			
Median	36	42	<0.001
Interquartile Range	30–44	36–49	
**Aboriginal ancestry, n (%)**			
No	216 (29)	1143 (39)	<0.001
Yes	188 (25)	247 (9)	
Unknown	341 (46)	1507 (52)	
**History of IDU, n (%)**			
No	243 (33)	1342 (46)	<0.001
Yes	432 (58)	954 (33)	
Unknown	70 (9)	601 (21)	
**System characteristics**			
**Prescriber experience (HIV patient caseload)**			
Median	96	108	<0.001
Interquartile Range	18–204	24–266	
**Place of residence at baseline, n (%)**			
Fraser	191 (26)	575 (20)	<0.001
Interior + Northern	118 (16)	237 (8)	
Vancouver Island	103 (14)	311 (11)	
Vancouver Coastal	328 (44)	1766 (61)	
**Year HAART was initiated, n (%)**			
2000–2003	234 (31)	874 (30)	0.552
2004–2007	271 (37)	1029 (36)	
2008–2010	240 (32)	994 (34)	

Non-compliance to HIV clinical care guidelines by gender are shown in **Table 2**. At baseline, 42% of women (vs. 34% of men (p<0.001)) did not receive drug resistance testing prior to starting treatment; 17% of women (vs. 9% of men (p<0.001)) started on a non-recommended ART regimen; and 47% of women (vs. 49% of men (p = 0.284)) started therapy with CD4 less than 200 cells/mm^3^. During the first year of follow-up, 17% of women (vs. 11% of men (p<0.001)) received less than 3 CD4 cell count tests; 17% of women (vs. 11% of men (p<0.001)) received less than 3 plasma viral load tests; and 50% of women (vs. 41% of men (p<0.001)) did not achieve HIV viral load suppression within 6 months of treatment initiation. Overall, QOC was better among men, who had a lower mean PCS score of 1.54 (SD = 1.30) compared with 1.89 (SD = 1.37) for women. Of note, only 14% of women received all six recommended care guidelines (vs. 23% of men), and 13% of women experienced non-compliance to 4 or more guidelines (vs. 9% of men) (p<0.001).

**Table 2:** Estimates of non-compliance with HIV clinical care guidelines by gender.γ

	Gender		
	Women (n = 745)	Men (n = 2897)	p-value
**Indicators at baseline**			
**(1) Not having drug resistance testing prior to starting treatment**			
Yes, n (%)	310 (42)	995 (34)	<0.001
No, n (%)	435 (58)	1902 (66)	
**(2) Starting on a non-recommended ART regimen**			
Yes, n (%)	126 (17)	256 (9)	<0.001
No, n (%)	619 (83)	2641 (91)	
**(3) Starting therapy with CD4 less than 200 cells/mm^3^**			
Yes, n (%)	347 (47)	1413 (49)	0.284
No, n (%)	398 (53)	1484 (51)	
**Indicators during the first-year of follow-up**			
**(4) Receiving less than 3 CD4 cell count tests**			
Yes, n (%)	124 (17)	312 (11)	<0.001
No, n (%)	621 (83)	2585 (89)	
**(5) Receiving less than 3 plasma viral load tests**			
Yes, n (%)	124 (17)	312 (11)	<0.001
No, n (%)	621 (83)	2585 (89)	
**(6) Not achieving HIV viral load suppression within six months of treatment initiation**			
Yes, n (%)	375 (50)	1178 (41)	<0.001
No, n (%)	370 (50)	1719 (59)	

γ Overall, QOC was better among men, who had a lower mean PCS score of 1.54 (SD = 1.30) compared with 1.89 (SD = 1.37) for women.

Over the past decade, individuals starting ART in more recent years were more likely to have a PCS score of 0, however progress in QOC was unequal by gender with the mean PCS score among men decreasing from 2.18 (SD = 1.26) in 2000 to 0.90 (SD = 1.05) in 2010, and 2.14 (SD = 1.33) to 1.31 (SD = 1.08) among women (data not shown, p-value for linear time trends for both genders <0.001).

In the multivariable ordinal logistic regression model (**Table 3**), female gender (unadjusted OR = 1.58 [95% CI: 1.37–1.83]; adjusted OR = 1.16 [95% CI: 0.99–1.35]; p = 0.062) was marginally associated with higher PCS scores (poorer QOC) after controlling for age, history of IDU, Aboriginal ancestry, and place of residence. Estimated model-based probabilities of PCS scores among women are shown in Table 4, with higher probabilities shown in bold. Among women, those with a history of IDU, of Aboriginal ancestry, from Vancouver Island, and who initiated ART in earlier years (2000–2003 and 2004–2007) were associated with a higher probability of worse PCS scores.

**Table 3:** Multivariable ordinal logistic regression model.

	Unadjusted OR (95% CI)	Adjusted* OR (95% CI)
**Gender (female vs. male)**	1.58 (1.37–1.83)	1.16 (0.99–1.35)

*Adjusted for: age, IDU, aboriginal ancestry, place of residence

**Table 4:** Estimated probabilities of PCS scores among women based on the results of multivariate non-proportional odds model

	Estimated probability of each PCS score (interquartile range)				
	PCS = 0	PCS = 1	PCS = 2	PCS = 3	PCS≥4
**Patient characteristics**					
**Aboriginal ancestry**					
No	0.11 (0.07,0.27)	**0.33 (0.31,0.41)**	0.27 (0.23,0.32)	0.12 (0.08,0.17)	0.08 (0.07,0.13)
Yes	0.04 (0.03,0.17)	0.23 (0.21,0.29)	**0.29 (0.28,0.32)**	0.18 (0.15,0.24)	0.22 (0.12,0.23)
Unknown	0.09 (0.05,0.23)	**0.32 (0.27,0.34)**	0.3 (0.25,0.31)	0.14 (0.1,0.18)	0.12 (0.08,0.17)
**History of IDU**					
No	0.13 (0.09,0.36)	**0.34 (0.33,0.39)**	0.27 (0.18,0.3)	0.1 (0.07,0.13)	0.08 (0.05,0.1)
Yes	0.06 (0.04,0.17)	0.28 (0.23,0.31)	**0.3 (0.28,0.32)**	0.17 (0.13,0.2)	0.16 (0.12,0.22)
Unknown	0.07 (0.06,0.22)	**0.33 (0.28,0.33)**	0.3 (0.25,0.32)	0.15 (0.12,0.19)	0.13 (0.09,0.15)
**System characteristics**					
**Place of residence at baseline**					
Fraser	0.11 (0.07,0.25)	**0.33 (0.31,0.38)**	0.29 (0.24,0.3)	0.12 (0.08,0.17)	0.1 (0.07,0.12)
Interior + Northern	0.07 (0.05,0.21)	**0.29 (0.25,0.33)**	**0.29 (0.26,0.32)**	0.15 (0.13,0.18)	0.12 (0.09,0.2)
Vancouver Island	0.05 (0.04,0.16)	0.25 (0.2,0.3)	**0.3 (0.28,0.31)**	0.17 (0.16,0.2)	0.2 (0.13,0.26)
Vancouver Coastal	0.08 (0.04,0.21)	**0.31 (0.25,0.34)**	0.29 (0.26,0.32)	0.14 (0.11,0.19)	0.13 (0.08,0.18)
**Year ART was initiated**					
2000–2003	0.05 (0.04,0.07)	0.28 (0.23,0.33)	**0.3 (0.29,0.3)**	0.21 (0.18,0.24)	0.16 (0.12,0.2)
2004–2007	0.07 (0.05,0.09)	0.3 (0.24,0.35)	**0.32 (0.31,0.33)**	0.14 (0.12,0.17)	0.16 (0.12,0.21)
2008–2010	0.27 (0.21,0.36)	**0.32 (0.31,0.34)**	0.23 (0.18,0.26)	0.1 (0.07,0.13)	0.07 (0.05,0.09)

The higher probabilities for each PSC score by patient and system characteristics are in bold.

## Discussion

The present study demonstrates that women were 58% more likely than men to receive poorer QOC, which is known to increase the risk of morbidity and mortality, and this association persisted (16%) after covariate adjustment. Further, non-receipt of recommended care was especially evident among women with a history of IDU, of Aboriginal ancestry, from Vancouver Island, and who initiated ART in earlier years. To our knowledge, this is the first long-term study to assess patient- and system-level factors associated with QOC during the first year on ART using a metric that predicts long-term health outcomes.

Poorer QOC observed among women in this study is consistent with other literature on this topic. Previous studies have shown that women have poorer access and adherence to ART than men [29], [30], [31]. Further, in the literature assessing performance on quality indicators in particular, one study also showed gender disparities in several elements of care, which persisted in clinics serving a high percentage of female clients [32]. Prior studies evaluating standards of care have identified various clinic, provider and patient level characteristics associated with quality indicators. Clinic factors found to be related to quality indicators include type of site (e.g., infection disease versus general medicine clinics) [33], experience with treating HIV patients [34], and integration of HIV and drug addiction services [35]. Provider experience, such as specialization, HIV patient caseload, and years of practice, has also been shown to be related to the receipt of indicated care [33], [34], [36], [37], [38], [39], [40], [41]. Patient characteristics such as ethnicity and substance use are also key predictors of adherence and quality of care [41]. Noteworthy, however, the indicators described in these studies are numerous and varied (indicating a need to streamline measures for assessing processes of care), and, unlike the PCS metric used here, are not correlated with actual health outcomes.

Overall, this study provides evidence that even within a fully subsidized health care system, there remain important gender inequities in access and adherence to necessary HIV treatment and care. These findings suggest that women still face several barriers to receipt of high-quality care. Our baseline gender comparisons and multivariate analyses suggest that experiences of drug use amongst other social determinants of health such as Aboriginal ancestry, which may create barriers to care, were more common among women than men in this cohort. This is an important finding that highlights the unique social realities women living with HIV face compared to men, underscoring the need for interventions that are responsive to these gendered realities. Further, even after accounting for these differences, female gender remained associated with poorer QOC in multivariate analyses, suggesting that there are other possible reasons for this gender gap in care that need to be addressed.

Potential barriers not explored in this analysis may include women's socio-economic status (e.g., income, education) [42], [43], intersectional stigma (e.g., sexism, racism, homophobia, classism, ableism, and HIV-related stigma),^17, 18^ gender-based violence and trauma [44], depression [45] and isolation [46], competing responsibilities (e.g., childcare) [47], [48], inflexibilities in clinic hours [49], [50], [51], negative experiences with health care providers [52], and a lack of services focusing on women's unique health and social concerns [53]. These intersecting and unequal social positions can affect HIV-positive women's health and access to healthcare [54], [55].

Research is limited regarding effective methods to reduce these gendered barriers and ensure women's equal access to and receipt of appropriate health care. However, the need for more tailored, gender-focused strategies that are responsive to women's needs is clear. While fairly underdeveloped in the HIV field, women-centred care (WCC) has been a recognized model for the provision of women's health care since the 1960s and 1970s [56]. According to a recent review that sought to conceptualize WCC in the HIV field, this approach to care, for example, often provides a women's majority environment with the option of seeing a female care provider; prioritizes a safe, non-judgemental atmosphere for care; provides transportation reimbursement, free childcare, food, and other specialized supports to address women's social and ancillary needs; is supportive of women's agency, empowerment, and active participation in their care; is attentive to women's diversity and lived experience and meets women where they are using a harm reduction model of care; and provides multidisciplinary service integration [57].

A model for WCC in BC is the Oak Tree Clinic, located in Vancouver. It has been a pioneer in implementing WCC for women and children living with HIV in BC. An ad hoc sub-analysis conducted comparing PCS scores between women who ever accessed care at Oak Tree Clinic during their first year on ART (n = 233) and women who did not (n = 509) found that Oak Tree patients were more likely to have lower PSC scores (better QOC), as shown in the Supplementary text (Tables S1 and S2). Further evaluation of the effectiveness of WCC models in improving health outcomes for women living with HIV is necessary.

There are limitations to this study. Firstly, the categorization of women versus men into groups was based on biological sex at birth, as data on self-reported gender identity was not available. Further, while transgender people were included in this analysis, they were also grouped according to biological sex at birth. Consequently, our conclusions do not accurately portray the health care experiences of all women in BC who may self-identify as a woman. Secondly, the patient and system characteristics included in this study were limited. Thus, the association between gender and QOC should be interpreted with caution, since it is possible that there are other confounding factors that were unmeasured and unadjusted for in this analysis. Efforts should be made to explore other possible reasons for gender inequities in QOC. Lastly, the DTP does not include data on patient pregnancy status, and, therefore, we were unable to determine which women may have started ART in the context of pregnancy. Further, some quality indicators in the PCS metric (such as resistance testing and classification of antiretroviral therapy regimens as recommended or not) do not take into account BC and Canadian care guidelines that are unique to pre-conception planning or pregnancy and postpartum treatment [58]. Of note for this study: (1) resistance testing, while recommended prior to treatment initiation for HIV-positive adults, is sometimes not possible in cases where therapy needs to be started rapidly for pregnancy or pre-pregnancy planning; and (2) Zidovudine (AZT), while no longer recommended as a first-line regime for HIV-positive adults, remains commonly prescribed to women who are pregnant or planning a pregnancy. As such, the PCS metric may be biasing some women towards poorer scores which may in part be explained by good clinical practice for HIV-positive pregnant women. Future gendered analyses must take into account issues that are unique to the care of HIV-positive pregnant women. Co-morbidities and mental health contribution to adherence and access to care should also be further explored.

Despite these limitations, there are also several strengths to this study. Firstly, the study utilized prospective, population-based cohort data of all individuals initiating ART in BC over a ten year time period. Further, the study was conducted within a universal healthcare system without user fees for care or ART medications, which means our findings are less likely to be biased by the confounding effect of financial barriers. Lastly, to our knowledge, this study uses a QOC metric that predicts long-term health outcomes, making it an important clinical benchmark for programmatic performance.

## Conclusions

High-quality HIV clinical care during the first year on ART is vital for long-term health and survival. However, the underlying social and structural barriers that undermine women's access to and maintenance of optimal treatment must be addressed in order for all women to experience improved health and well-being. The expansion of ART programs now underway not only in BC but around the world provides a timely opportunity to reduce these gender inequities in QOC. Patients need to be informed about gender-specific treatment initiation guidelines and the implications of poor compliance. Administrative bodies need to monitor PCS quality indicators and provide health providers with evaluative feedback. This study also demonstrates an urgent a need for women-centred models of care such as Oak Tree Clinic that acknowledge and address the gendered barriers to HIV treatment for women living with HIV. Additional evaluation studies on WCC are necessary, and further research is needed to understand barriers to care from a gendered perspective. This will be conducted as part of the Canadian HIV Women's Sexual and Reproductive Health Cohort Study (CHIWOS; www.chiwos.ca), a new prospective cohort study of 1,250 HIV-positive women in Canada. This study will have important implications for gender-sensitive and culturally-appropriate models of care for women living with HIV in Canada and globally, which have the potential to both improve individual health outcomes and reduce risks of HIV transmission.

## Supporting Information

Figure S1 **Recommended regimens based on the IAS guidelines for treating HIV-positive adults between 2000 and 2010.**
(DOC)

Table S1 **Estimated probabilities of PCS scores among women who ever accessed Oak Tree Clinic during their first year on HAART (n = 233) and women who did not (n = 509) based on the results of multivariate non-proportional odds model.**
(DOC)

Table S2 **Adjusted odds ratios showing factors associated with poorer QOC among women who ever accessed Oak Tree Clinic during their first year on HAART (n = 233) and women who did not (n = 509) based on the results of multivariate non-proportional odds model.**
(DOC)
