# Supplementary material for: Gender Inequities in Quality of Care among HIV-Positive Individuals Initiating Antiretroviral Treatment in British Columbia, Canada (2000–2010)
Source: PLoS One. 2014 Mar 18;9(3):e92334. doi: 10.1371/journal.pone.0092334 (PMC3958538; doi:10.1371/journal.pone.0092334)
Supplement: Figure S1 — Recommended regimens based on the IAS guidelines for treating HIV-positive adults between 2000 and 2010. (DOC) [file pone.0092334.s001.doc]

**Figure S1.** Recommended regimens based on the IAS guidelines for treating HIV-positive adults between 2000 and 2010

**2000-2003:**

- Two comparable nucleosides, or a nucleoside and a nucleotide reverse transcriptase inhibitor plus either (1) a non-nucleoside reverse transcriptase inhibitor, or (2) a protease inhibitor boosted with ritonavir, or (3) a single protease inhibitor;
- Three nucleoside reverse transcriptase inhibitors if:
- zidovudine + lamivudine + abacavir; or
- zidovudine + didanosine + lamivudine; or
- lamivudine + stavudine + abacavir.

**2004-2005:**

- Two nucleoside reverse transcriptase inhibitors if:
- zidovudine or tenofovir + (lamivudine or emtricitabine); or
- emtricitabine + didanosine.
- *Plus either:*
- efavirenz or nevirapine; or
- lopinavir, atazanavir, indinavir or saquinavir boosted w/ ritonavir.
- nelfinavir

**2006-2008:**

- Two nucleoside reverse transcriptase inhibitors if:
- emtricitabine + tenofovir; or
- (abacavir or zidovudine) + lamivudine; or
- tenofovir + lamivudine
- *Plus either:*
- efavirenz or nevirapine;
- lopinavir, atazanavir, amprenavir, fosamprenavir or darunavir boosted w/ ritonavir.

**2008-2010:**

- Two nucleoside reverse transcriptase inhibitors if:
- emtricitabine + tenofovir; or
- abacavir + lamivudine
- *Plus either:*
- efavirenz or nevirapine; or
- lopinavir, atazanavir, amprenavir, fosamprenavir or darunavir boosted w/ ritonavir; or
- maraviroc
