# Supplementary material for: Gender Inequities in Quality of Care among HIV-Positive Individuals Initiating Antiretroviral Treatment in British Columbia, Canada (2000–2010)
Source: PLoS One. 2014 Mar 18;9(3):e92334. doi: 10.1371/journal.pone.0092334 (PMC3958538; doi:10.1371/journal.pone.0092334)
Supplement: Table S1 — Estimated probabilities of PCS scores among women who ever accessed Oak Tree Clinic during their first year on HAART (n = 233) and women who did not (n = 509) based on the results of multivariate non-proportional odds model. (DOC) [file pone.0092334.s002.doc]

**Table SI. Estimated probabilities of PCS scores among women who ever accessed Oak Tree Clinic during their first year on HAART (n=233) and women who did not (n=509) based on the results of multivariate non-proportional odds model**

|  | **Estimated probability of each PCS score (interquartile range)** | | | | |
| --- | --- | --- | --- | --- | --- |
|  | **PCS=0** | **PCS=1** | **PCS=2** | **PCS=3** | **PCS≥4** |
| **Patient characteristics** |  |  |  |  |  |
| **Aboriginal ancestry** |  |  |  |  |  |
| No | 0.1 (0.08,0.29) | **0.29 (0.28,0.38)** | **0.3 (0.22,0.32)** | 0.13 (0.09,0.15) | 0.09 (0.06,0.14) |
| Yes | 0.05 (0.04,0.17) | 0.25 (0.18,0.29) | **0.29 (0.28,0.32)** | 0.19 (0.15,0.21) | 0.19 (0.12,0.25) |
| Unknown | 0.07 (0.05,0.24) | **0.29 (0.24,0.4)** | **0.29 (0.26,0.31)** | 0.13 (0.09,0.18) | 0.12 (0.08,0.18) |
| **History of IDU** |  |  |  |  |  |
| No | 0.13 (0.09,0.33) | **0.35 (0.29,0.46)** | 0.26 (0.21,0.3) | 0.1 (0.07,0.13) | 0.08 (0.05,0.09) |
| Yes | 0.06 (0.04,0.18) | 0.26 (0.2,0.29) | **0.31 (0.28,0.32)** | 0.17 (0.13,0.2) | 0.15 (0.12,0.21) |
| Unknown | 0.06 (0.05,0.2) | **0.32 (0.26,0.41)** | 0.3 (0.25,0.31) | 0.14 (0.11,0.2) | 0.12 (0.08,0.17) |
| **System characteristics** |  |  |  |  |  |
| **Place of residence at baseline** |  |  |  |  |  |
| Fraser | 0.1 (0.07,0.27) | **0.32 (0.28,0.46)** | **0.29 (0.21,0.32)** | 0.12 (0.08,0.16) | 0.08 (0.06,0.13) |
| Interior + Northern | 0.09 (0.05,0.24) | **0.27 (0.25,0.3)** | **0.29 (0.27,0.32)** | 0.14 (0.13,0.18) | 0.12 (0.09,0.19) |
| Vancouver Island | 0.06 (0.04,0.17) | 0.24 (0.18,0.28) | **0.3 (0.28,0.32)** | 0.17 (0.15,0.21) | 0.18 (0.13,0.25) |
| Vancouver Coastal | 0.08 (0.05,0.2) | **0.29 (0.23,0.37)** | **0.3 (0.26,0.32)** | 0.14 (0.1,0.19) | 0.12 (0.08,0.19) |
| **Year ART was initiated** |  |  |  |  |  |
| 2000-2003 | 0.05 (0.04,0.07) | 0.28 (0.2,0.36) | **0.3 (0.28,0.31)** | 0.21 (0.16,0.24) | 0.16 (0.11,0.19) |
| 2004-2007 | 0.06 (0.05,0.09) | 0.29 (0.22,0.37) | **0.32 (0.3,0.34)** | 0.14 (0.11,0.18) | 0.15 (0.12,0.21) |
| 2008-2010 | 0.27 (0.21,0.35) | **0.29 (0.27,0.29)** | 0.22 (0.18,0.27) | 0.09 (0.07,0.13) | 0.07 (0.05,0.09) |
| **Ever Oak Tree Clinic** |  |  |  |  |  |
| No | 0.09 (0.05,0.24) | 0.27 (0.2,0.29) | **0.3 (0.27,0.32)** | 0.15 (0.12,0.2) | 0.13 (0.08,0.2) |
| Yes | 0.07 (0.04,0.13) | **0.43 (0.35,0.46)** | 0.27 (0.22,0.3) | 0.11 (0.08,0.14) | 0.09 (0.06,0.14) |

The higher probabilities for each PSC score by patient and system characteristics are in bold.
