# Supplementary material for: Gender Inequities in Quality of Care among HIV-Positive Individuals Initiating Antiretroviral Treatment in British Columbia, Canada (2000–2010)
Source: PLoS One. 2014 Mar 18;9(3):e92334. doi: 10.1371/journal.pone.0092334 (PMC3958538; doi:10.1371/journal.pone.0092334)
Supplement: Table S2 — Adjusted odds ratios showing factors associated with poorer QOC among women who ever accessed Oak Tree Clinic during their first year on HAART (n = 233) and women who did not (n = 509) based on the results of multivariate non-proportional odds model. (DOC) [file pone.0092334.s003.doc]

**Table SII.** Adjusted odds ratios showing factors associated with poorer QOC among women who ever accessed Oak Tree Clinic during their first year on HAART (n=233) and women who did not (n=509) based on the results of multivariate non-proportional odds model

| **Variable** | **Odds ratio** | | | **P-value** |
| --- | --- | --- | --- | --- |
|  | **Estimate** | **95% CI** | |  |
| **Patient characteristics** |  |  |  |  |
| **Aboriginal** |  |  |  |  |
| No | 1.000 |  |  |  |
| Yes | 2.036 | 1.396 | 2.969 | **<.0001** |
| Unknown | 1.498 | 1.084 | 2.068 | **0.014** |
| **IDU** |  |  |  |  |
| No | 1.000 |  |  |  |
| Yes | 1.734 | 1.283 | 2.343 | **<.0001** |
| Unknown | 1.469 | 0.894 | 2.414 | 0.129 |
| **System characteristics** |  |  |  |  |
| **Place of residence at baseline** |  |  |  |  |
| Fraser | 0.935 | 0.673 | 1.301 | 0.692 |
| Interior and Northern | 1.075 | 0.713 | 1.622 | 0.729 |
| Vancouver Island | 1.556 | 1.040 | 2.327 | **0.032** |
| Vancouver Coastal | 1.000 |  |  |  |
| **Year ART was initiated** |  |  |  |  |
| 2000-2003 (vs. 2008-2010) |  |  |  |  |
| PCS ≥1 vs. 0 | 6.473 | 3.439 | 12.184 | **<.0001** |
| PCS ≥2 vs. ≤1 | 3.326 | 2.238 | 4.943 | **<.0001** |
| PCS ≥3 vs. ≤2 | 2.986 | 1.921 | 4.643 | **<.0001** |
| PCS ≥4 vs. ≤3 | 2.481 | 1.354 | 4.547 | **0.003** |
| 2004-2007 (vs. 2008-2010) |  |  |  |  |
| PCS ≥1 vs. 0 | 4.941 | 2.885 | 8.462 | **<.0001** |
| PCS ≥2 vs. ≤1 | 2.720 | 1.856 | 3.987 | **<.0001** |
| PCS ≥3 vs. ≤2 | 2.228 | 1.440 | 3.450 | **<.0001** |
| PCS ≥4 vs. ≤3 | 2.543 | 1.411 | 4.583 | **0.002** |
| **Ever Oak Tree Clinic (No vs. Yes)** |  |  |  |  |
| PCS ≥1 vs. 0 | 0.793 | 0.482 | 1.307 | 0.363 |
| PCS ≥2 vs. ≤1 | 1.787 | 1.255 | 2.544 | **0.001** |
| PCS ≥3 vs. ≤2 | 1.643 | 1.100 | 2.456 | **0.015** |
| PCS ≥4 vs. ≤3 | 1.470 | 0.886 | 2.438 | 0.136 |
